# Supplementary figures and images for: Rapid adaptation to human protein kinase R by a unique genomic rearrangement in rhesus cytomegalovirus
Source: PLoS Pathog. 2021 Jan 26;17(1):e1009088. doi: 10.1371/journal.ppat.1009088 (PMC7864422; doi:10.1371/journal.ppat.1009088)

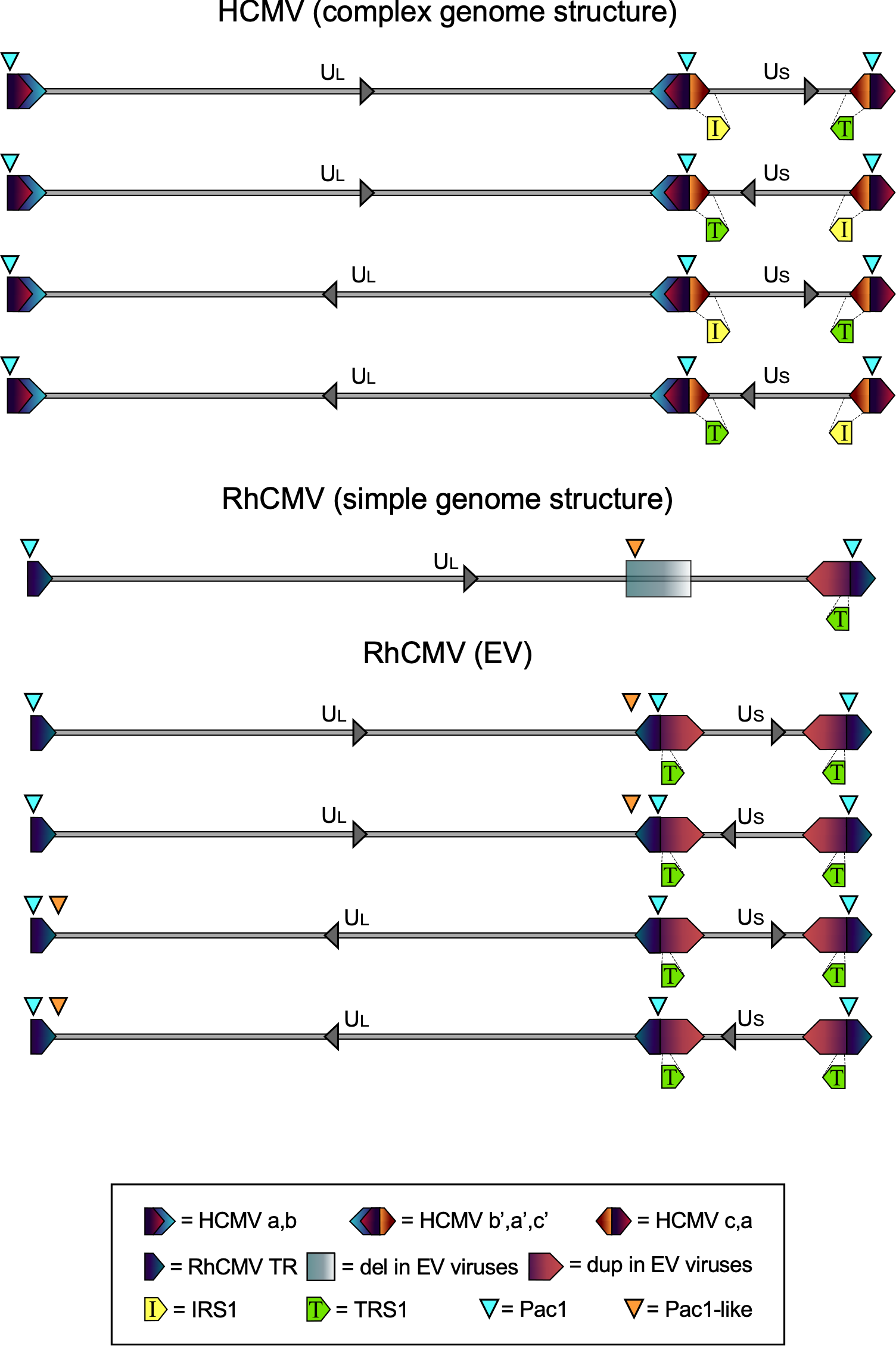

Supplement: S1 Fig — The inverted duplication of the end of the RhCMV genome, including the terminal repeat, that generated the evolved viruses reported in this manuscript, results in a type E genomic organization that is very similar to that found in HCMV as well other hominoid and New World Monkey CMVs. The parental RhCMV, like other Old World monkey CMVs, has a type A genomic structure with direct terminal repeats but no internal repeat sequences. (TIF) [file ppat.1009088.s001.tif]
