# Supplementary material for: Rapid adaptation to human protein kinase R by a unique genomic rearrangement in rhesus cytomegalovirus
Source: PLoS Pathog. 2021 Jan 26;17(1):e1009088. doi: 10.1371/journal.ppat.1009088 (PMC7864422; doi:10.1371/journal.ppat.1009088)
Supplement: Attachment — Submitted filename: Response to reviewers 12.31.2020 resubmission.docx [file ppat.1009088.s002.docx]

December 31, 2020

Dear Editors,

We thank the editors and reviewers for their careful review of our paper. We have revised the manuscript to address the issues raised as follows:

**Part II – Major Issues: Key Experiments Required for Acceptance**

**Comment 1: To better highlight the proposed Pac-based duplication and E class isomerizations of genomes, a diagram illustrating hCMV isomers might be added to make a more direct comparison to RhCMV. This is partially illustrated in Figure 6, but missing the hCMV comparison, which might be developed further. For example, is TRS1/IRS1 in hCMV a likely outcome of a similar duplication? Might it be possible to get hCMV to “revert” to a more simple single isomer based on the RhCMV result in the other direction? Is there any phylogenetic evidence that the Pac sequence arrangements found in RhCMV are ancestral?**

Response: We have added a supplemental figure (S1 Fig.) comparing HCMV and RhCMV genomic structures, which illustrates this point. We refer to this figure (lines 202-205, 222 and 387-389) and discuss the idea that TRS1 and IRS1 may have resulted from an ancient duplication (lines 330-332).

We suspect that HCMV could revert to a simple form. Perhaps the ancestral Old World monkey CMVs had a full internal repeat (E genome structure) that underwent such a process. However, there are no actual data to support this idea and experimentally approaching this issue is likely to be difficult. Since many HCMV genes are not necessary for replication in cell culture and yet are retained in the genome, it could be difficult to observe loss of the duplicated copy of rTRS1 just by removing the PKR selective pressure.

**Comment 2: In some of the follow-up experiments the authors describe experimental evolution of RhCMV in rhesus fibroblasts and human fibroblasts lacking PKR. Did the viruses improve in ability to replicate after these protocols?**

Response: We found that the evolved viruses do tend to replicate slightly less well in HF^ΔPKR^ than in wt HF (Fig. 2 and text lines 131-139). Whether or not they replicate better in RF is an interesting experiment but we feel it is beyond the scope of this report.

**Comment 3: Is EV3 better at blocking phosphorylation of PKR and eIF2alpha compared to EV1 and EV2? Are the blots (westerns and southerns) representative of multiple experiments?**

Response: The quantitative measures of phosphorylation of PKR and eIF2alpha were not precise enough to allow us to conclude that EV3 is a better blocker than EV1 and EV2 (lines 251-254). We did repeat the westerns and Southern blots at least once each with qualitatively similar results, as we now state in the legends to Figs. 6-8.

**Comment 4. The data are consistent with TRS1 gene dosage via coding region duplication being the primary selected phenotype that allows enhanced growth on HF. However, given that other genomic changes occurred and their effects may also make contributions as well as the fact that growth is not totally restored on PKR-deficient cells, additional measures should be pursued to establish the specific necessity of TRS1-PKR relationship. For instance, demonstrating that ectopic overexpression of TRS1 rescues growth of parental RhCMV on HF would enhance the strength of this conclusion. Alternatively, perhaps it’s possible to demonstrate that during coinfection of HF with evolved and parental RhCMV allowed enhanced growth of the latter (as indicated by genomic DNA qPCR using strain-specific primers).**

Response: The first suggested experiment is a logical one but we think it is not critical to support our key findings. We have shown previously in two other systems, using recombinant VACVs and HCMVs in which PKR antagonism depends on a single copy of rTRS1, that additional expression of rTRS1 from a cellular transgene rescues replication (Brennan et al, 2014 and Child et al 2018). We discuss these results more clearly now in (lines 240-243). The alternative suggested experiment is potentially interesting but co-infection of cells with HCMV and RhCMV is likely to be involve multiple complex virus-virus interactions that could confound any clear conclusions.

**Comment 5. To demonstrate the specificity of the PKR evasion phenotype as functionally important to enhanced growth on HF (as opposed to evasion of other HF-specific factors), the virus strain that was evolved on PKR-deficient HF should be used as a control in virus replication experiments as in Fig. 2C.**

Response: The fact that we did not detect any high frequency mutations in the viruses that were passaged in HF^∆PKR^ means there is not a compelling reason to suspect these passaged viruses would have any phenotypic differences from the parent virus (lines 191-193).

**Comment 6. It is important to quantify the specific increase of TRS1 expression in the evolved relative to the parental viruses and perhaps even examine the correlation of this with the degree of growth enhancement. Ideally this would be done by qRT-PCR but at a minimum could be done by image pixel quantitation of Fig. 7.**

Response: We did quantify the expression of rTRS1 after infection with these viruses, as shown at the bottom in the blot in Fig. 7.

**Comment 7. The authors should explain why 3 bands are evident in the TRS1 immunoblot image in Fig. 7.**

Response: We do not know why we see multiple bands in the TRS1 blot, but we have observed this previously as well (Ref 21). We suspect they may result from posttranslational modifications of the protein, as we now note in lines 236-238.

**Part III – Minor Issues: Editorial and Data Presentation Modifications.**

**Comment 8: In several places the authors mention increases in virus fitness, but this is narrowly defined in reference to human fibroblast infections in the presence of PKR, whereas the accompanying deletions lead to potentially worse replication cells in lacking PKR and also likely in natural animal infections given details of the virus genes deleted in cell-adapted isolates. It might be easiest to modify the language to be more precise about gains in virus replication versus the more loaded term “fitness”. Related to this point, how well do EV1, EV2, and EV3 viruses replicate in rhesus fibroblasts?**

Response: We agree and now use the term “fitness” more sparingly. Testing how well EV1, EV2 and EV3 replicate in rhesus fibroblasts will be of interest and will be part of our ongoing project aiming to understand whether multiple PKR antagonists provide a benefit in different conditions, such as after exposure to interferon. However, the interpretation of these data may be complicated by the potential impact of the other gene duplications and deletions in these viruses.

**Comment 9: I couldn’t find any details about the serial infection protocols for experimental evolution besides the general schematic in Figure 2. Important details seem to be missing from the legend and/or methods section.**

Response: We have added more details about the experimental evolution protocol in the methods section (lines 423-431).

**Comment 10. Consider showing actual datapoints instead of bar graphs in Figures 1 and 2.**

Response: We have modified Figures 1 and 2 as suggested.

**Comment 11. How much read depth was obtained on the parental stocks to determine if the TRS1 duplication events are already present in rare genomes?**

Response: We now report that among the total reads (~ 1500X coverage) of our parent stock, we detected one read that may be due to this rearranged L junction in the parent stock. We discuss this issue in lines 358-374.

**Comment 12. Would the combinations of deep sequencing and Sanger based analysis reveal if there are some virus genomes replicating with more than 2 copies of rTRS1?**

Response: Our deep sequencing data show that the average rTRS1 copy number is close to two and we did not identify other junctions around rTRS1, so we think there is not a major population of viruses with more than two TRS1 copies. However, we acknowledge that we cannot be sure that there isn’t some heterogeneity in the number of copies in each genome (lines 281-283).

**Comment 13. To more definitively link the Pac sequences to the recombination process it would be ideal to have a virus strain with altered or deleted Pac sites to test whether they promote recombination under selection of human cells. Such experiments are not required in a revision for this reviewer, but might be mentioned as a future direction if the authors agree that this is a feasible and useful experiment**.

Response: We agree and now note that further study of the role of Pac1 in recombination is worthwhile (lines 181-184). In fact, we have a preliminary finding that pac1-like is used as a site for insertion of a horizontal gene transfer event; However, we feel this observation is premature to report at this time.

**Comment 14. I would consider modifying the title of the manuscript to highlight the isomerization of RhCMV to resemble hCMV through a single inverted duplication event.**

Response: While we agree that the similarity in the isomerizing ability of our evolved RhCMV to HCMV is one of our interesting findings, we think adding that point would make the title too long and complex. However, we do now discuss this point more thoroughly in the text (e.g. lines 202-209) and emphasize it by inclusion of the new supplemental figure (Fig. S1).

**Technical Check comment: We noticed that you used “data not shown” in the manuscript, we do not allow these references. The PLOS data access policy requires that all data be either published with the manuscript or made available in a publicly accessible database.**

We have removed “data not shown” from the two places where we had used it in the original submission. In both instance, the relevant data are now available in the sequencing data that we have been deposited in the NCBI.

Thank you for considering our revised manuscript.

Sincerely,

Adam P. Geballe, M.D.

Professor and Associate Director, Division of Human Biology, Fred Hutchinson CRC

Professor, Clinical Research Division, Fred Hutchinson CRC

Professor, Departments of Medicine and Microbiology (adjunct), University of Washington
